# A novel phantom technique for evaluating the performance of PET auto-segmentation methods in delineating heterogeneous and irregular lesions

**DOI:** 10.1186/s40658-015-0116-1

**Published:** 2015-06-27

**Authors:** B Berthon, C Marshall, R Holmes, E Spezi

**Affiliations:** Wales Research and Diagnostic Positron Emission Tomography Imaging Centre, Cardiff University - PETIC, room GF705 Ground floor ‘C’ Block, Heath Park, CF14 4XN Cardiff, UK; Department of Medical Physics and Bioengineering, University Hospitals Bristol, BS2 8HW Bristol, UK; School of Engineering, Cardiff University, Cardiff, Wales UK

**Keywords:** Positron emission tomography, ^18^F-fluorodeoxyglucose, Imaging phantoms, Image segmentation, Inkjet printing, Radiotherapy

## Abstract

**Background:**

Positron Emission Tomography (PET)-based automatic segmentation (PET-AS) methods can improve tumour delineation for radiotherapy treatment planning, particularly for Head and Neck (H&N) cancer. Thorough validation of PET-AS on relevant data is currently needed. Printed subresolution sandwich (SS) phantoms allow modelling heterogeneous and irregular tracer uptake, while providing reference uptake data. This work aimed to demonstrate the usefulness of the printed SS phantom technique in recreating complex realistic H&N radiotracer uptake for evaluating several PET-AS methods.

**Methods:**

Ten SS phantoms were built from printouts representing 2mm-spaced slices of modelled H&N uptake, printed using black ink mixed with 18F-fluorodeoxyglucose, and stacked between 2mm thick plastic sheets. Spherical lesions were modelled for two contrasted uptake levels, and irregular and spheroidal tumours were modelled for homogeneous, and heterogeneous uptake including necrotic patterns. The PET scans acquired were segmented with ten custom PET-AS methods: adaptive iterative thresholding (AT), region growing, clustering applied to 2 to 8 clusters, and watershed transform-based segmentation. The difference between the resulting contours and the ground truth from the image template was evaluated using the Dice Similarity Coefficient (DSC), Sensitivity and Positive Predictive value.

**Results:**

Realistic H&N images were obtained within 90 min of preparation. The sensitivity of binary PET-AS and clustering using small numbers of clusters dropped for highly heterogeneous spheres. The accuracy of PET-AS methods dropped between 4% and 68% for irregular lesions compared to spheres of the same volume. For each geometry and uptake modelled with the SS phantoms, we report the number of clusters resulting in optimal segmentation. Radioisotope distributions representing necrotic uptakes proved most challenging for most methods. Two PET-AS methods did not include the necrotic region in the segmented volume.

**Conclusions:**

Printed SS phantoms allowed identifying advantages and drawbacks of the different methods, determining the most robust PET-AS for the segmentation of heterogeneities and complex geometries, and quantifying differences across methods in the delineation of necrotic lesions. The printed SS phantom technique provides key advantages in the development and evaluation of PET segmentation methods and has a future in the field of radioisotope imaging.

## Background

Positron emission tomography (PET) imaging using ^18^F-fluorodeoxyglucose (^18^F-FDG) allows the observation of metabolic pathways in the human body and is therefore increasingly used for gross tumour volume (GTV) delineation for a number of cancers, including head and neck (H&N). The use of PET-based automatic segmentation (PET-AS) methods could be useful in radiotherapy treatment planning and in the prediction of response to therapy, for which accurate segmentation of the tumours is crucial. Some studies have shown that PET-AS methods which perform well with homogeneous lesions show poor accuracy in the case of more realistic inhomogeneous and irregular clinical lesions, using clinical or simulated data [1, 2], in particular when using fixed thresholding methods, which are highly dependent on the image type [3]. The use of advanced PET-AS beyond thresholding was recommended to reduce dosimetry errors, especially in the case of heterogeneous tumours [4]. Although an increasingly large number of studies have investigated and compared the performance of existing PET segmentation methods, the target objects used are most frequently obtained with plastic fillable phantoms, including inserts of spherical geometry [5, 6]. Plastic phantoms combine the advantage of a known ground truth and a physical object, which can be scanned using patient protocols. However, these phantoms are limited to modelling simplified and clinically unrealistic uptake patterns. Furthermore, due to their fixed regular geometry, they do not allow modelling intra-tumour heterogeneity, which is a key element of clinical lesions. In addition, we have shown in a previous work that the presence of thick plastic walls encompassing the target object has an important effect on the evaluation of PET-AS methods [7]. Therefore, such phantoms are not adequate for studies requiring accurate modelling of patient metabolic uptake [8, 9], particularly in the H&N where the intricate anatomy and heterogeneity occurring in both background and tumour make the task of delineating the GTV very challenging. A small number of phantom studies have used deformed objects or molecular sieves to model non-spherical lesions [10–13] or have included absorbent material into their inserts to model inhomogeneities [14]. However, these techniques did not allow modelling combined heterogeneity and geometrical complexity in a controlled and reproducible manner and most still included the presence of glass or plastic walls. To our knowledge, heterogeneity and complex geometry have not yet been modelled in combination in realistic phantoms.

The use of printed radioactive uptake patterns has been investigated in the literature as a promising technique for generating radioactive sources for PET [15–17]. This allows modelling any desired tracer distribution while providing reference data or ground truth useful for a number of quality assurance purposes. A quantitative calibration study of the printing method was described in detail by Markiewicz et al*.* [17] for generating single-slice patterns with applications to brain imaging studies. However, the stacking of several printed patterns to produce a 3D object for quantitative applications was not investigated. Recent work by Holmes et al. used a 3D-printed phantom, named subresolution sandwich (SS) phantom, for the generation of realistic SPECT brain images [18]. However, to our knowledge, the use of stacked ^18^F-FDG-printed uptake patterns to generate a 3D PET phantom has not yet been investigated nor used for the evaluation of PET segmentation techniques.

This work aimed at demonstrating the advantages of using irregular and heterogeneous target objects to evaluate and compare the performance of PET-AS methods. For this purpose, we calibrated and used a novel 3D-printed SS phantom technique to acquire realistic image data. We used the PET images obtained by scanning the 3D-printed SS phantoms to evaluate and compare a set of ten PET-AS methods representing different medical image segmentation approaches. We have investigated the benefits of using the printed SS phantom compared to a standard plastic fillable phantom for testing PET-AS methods intended for radiotherapy treatment planning.

## Methods

### Experimental method and reproducibility

#### Preparation of the SS phantom

**Fig. 1:**
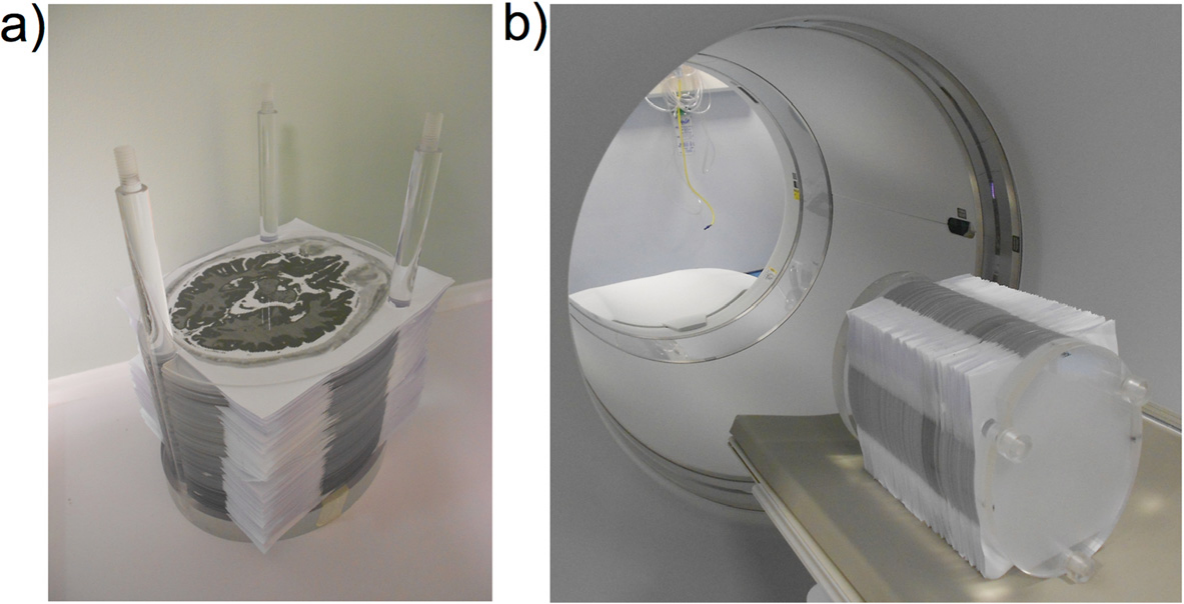
**a** Partially assembled printed SS phantom and **b** assembled phantom positioned on the scanner bed

The printed SS phantom structure consists of 120 oval poly(methyl methacrylate) (PMMA) sheet of 2-mm thickness, corresponding to axial slices, which can be assembled using three plastic rods attached to a cylindrical PMMA support. The radioactive part of the phantom, when containing radioactive printouts, can reach a maximum length of 240 mm. The paper and PMMA are held together by a thick plastic sheet, which is screwed on top of the phantom once assembled, allowing it to be scanned as a 3D physical object. A picture of the assembled 3D phantom is shown on Fig. 1a, along with the position of the phantom in the scanner on Fig. 1b.

Plain A4 80-mg paper was used, cut to 168 mm × 197 mm to fit into the phantom and hole punched in order for it to be assembled on the rods. Uptake printouts were generated as grey-level 3D images in Matlab (The MathWorks Inc., Natick, USA), resampled to 2-mm slices and printed on a HP deskjet 990 cxi, using drop-on-demand thermal inkjet printing. The advantage of this type of equipment is its use of refillable ink cartridges, making it possible to add the desired quantity of radiotracer to the same cartridge before each set of experiments. The printing settings “normal” and “black & white” were chosen in order to minimise the printing time (and therefore the radiotracer decay and user exposure to gamma emissions) while ensuring a good printing quality. The corresponding printing speed is 6.5 pages per minute. The printing resolution used throughout this work was 600 × 600 dpi.

The cartridge was filled with the desired ^18^F-FDG volume and topped with black ink. Various ^18^F-FDG activity concentrations were used for the different experiments. The images were printed in a hot cell (Gravatom Engineering Systems Ltd, Southampton, UK), after leaving the cartridge with its dispensing head down for 20 min to homogenize its contents, as recommended by the manufacturer. All operations including filling the ink cartridge and assembling the phantom were done behind a lead glass shield (Bright Technologies Ltd, Sheffield, UK). Any inaccuracy in the positioning of the pattern on the paper was corrected for by aligning markers printed as part of the pattern to reference markers drawn on the PMMA sheet. The cross-shaped markers were printed with the same radioactive ink as the printout and were visible on the PET image obtained. The phantom was scanned immediately after assembling on a GE 690 Discovery PET/CT scanner for two bed positions with the protocol used for clinical whole body diagnostic scans, given in Table 1. Both low-dose CT (used for attenuation correction) and high-resolution CT were acquired. Operator exposure to the radioactive tracer was controlled using standard safety equipment (e.g. lead glass shields, shielded syringe carriers, hot cell) and monitored with electronic portable dosimeters (RAD-60S, RADOS Technology, Oy, Finland). We assessed the homogeneity and reproducibility of the printing to ensure reliable printing of the desired uptake distributions.

**Table 1:** Parameters used for the acquisition and reconstruction of PET scans

Parameter	Value
2D matrix size CT (voxels)	512 × 512
2D matrix size PET (voxels)	256 × 256
Voxel size high resolution CT	0.977 mm × 0.977 mm × 2.5 mm
Voxel size PET	2.73 mm × 2.73 mm × 3.27 mm
Field of view dimensions	700 mm × 153 mm
Duration of bed position	3 min
Reconstruction algorithm	Vue Point FX TOF-corrected
Algorithm settings	3D ML OSEM 24 subsets 2 iterations
Post-processing filter cut-off	6.4 mm
CT-based attenuation correction	yes

The printing, assembling and scanning of the SS phantom took approximately 80 min for each experiment. This included (a) filling the cartridge (10 min), (b) leaving the contents of the cartridge to homogenize (10 min), (c) printing (30 min), (d) assembling (20 min) and (e) scanning (10 min). The whole body radiation dose to the operator for one session with a single scan was 4 μSv.

#### Printing quality

To assess the printing homogeneity, we printed two 30 mm × 200 mm stripes with a mixture of black ink and radiotracer along both width and length of an A4 paper. The number of counts was measured along these stripes, using thin layer chromatography (TLC) (iScan, Canberra, Uppsala, Sweden) at a speed of 1 mm/s.

The printing reproducibility was assessed using a 100 × 100 mm homogeneous square. This was printed with the same grey level and radioactive ink mixture 66 consecutive times. The phantom obtained by stacking these printouts was then scanned, and the resulting PET image was analysed. A region of interest (ROI) positioned at the centre of each square was reproduced on 60 consecutive slices (the superior and inferior edges of the phantom were excluded) of the PET image and the mean intensity of each ROI was measured.

#### Printer calibration

Additional experiments aimed at determining the relationship between grey levels specified to the printer and obtained on the PET image and derive an adequate calibration to ensure that the desired tissue uptake ratios were carried out. In this case, ten grey levels ranging from 10 to 100 % of the maximum printed intensity were defined and for each grey level, a 140 mm × 160 mm homogeneous rectangle was printed five times with the same mixture of black ink and ^18^F-FDG. The paper was weighed before and after printing to measure the amount of ink added by the printer. The weight of ink printed for each grey level, averaged over the five instances, was then plotted against the grey-level values specified. Furthermore, 20 distinct homogeneous 30 mm × 30 mm squares of grey-level values evenly spaced within 5 and 100 % were printed with the radioactive ink mixture. The number of counts detected across the different rectangles was then measured using the iScan TLC. Correction for radioactive decay was applied to compare all readings at the same time point. This process was repeated with three different activity concentrations in the ink at the time of measurement corresponding to different volumes of black ink added to 2 mL of the same radiotracer solution. The relationship between counts and the amount of ink printed on the paper was then derived.

In all experiments, the accuracy of the paper positioning in the phantom was assessed using radioactive cross-shaped markers printed at the top (T), left (L) and right (R) of the printout. The markers’ position on the acquired PET image was determined for each slice, as the highest intensity voxel in a 5 × 5 voxel square drawn around the imaged marker. For each one of the T, L and R markers, the difference in positioning with the average marker position was measured.

### Generation of realistic 3D uptake maps

**Fig. 2:**
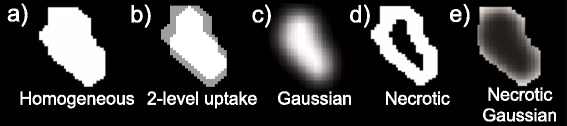
Modelled tumour patterns shown in a transverse slice of the irregular lesion. **a** Homogeneous. **b** 2-level uptake. **c** Gaussian. **d** Necrotic. **e** Necrotic Gaussian

A first uptake map was generated to model six spherical tumours of diameters 10, 13, 17, 22, 28 and 38 mm, named S1, S2, S3, S4, S5 and S6, respectively, with two levels of intensity, with the difference between the highest (central) uptake and lowest uptake equal to the difference between the lowest tumour uptake and background. This uptake pattern is shown on Fig. 2b. The methods described in the next section were applied to the six images obtained.

We further aimed at using the printed SS phantom to generate realistic irregular and heterogeneous target lesions. For this purpose, a clinical tumour outline was extracted from an available H&N PET/CT scan using manual delineation. The background uptake was modelled by segmenting normal anatomical structures on the CT scan and assigning to each structure a grey-level value corresponding to its mean ^18^F-FDG uptake, measured on the PET image. Ellipsoidal outlines were also used for different experiments at the same locations as the irregular tumour outlines on the background printout template. These target lesions were modelled with a volume of 11 mL, which is large enough to allow better investigation of highly heterogeneous uptake patterns, such as necrotic centres encountered in large lymph nodes. The different images printed corresponded to the background image, in which one of the volumes (irregular tumour or ellipsoid) was inserted with a grey-level value representing the desired ^18^F-FDG uptake. The resulting templates were resampled to 2-mm slices in the superior-inferior direction of the H&N scan, in order to match the thickness of the PMMA sheets. This process allowed the retrieval of the modelled tumour contour from the final printout template, providing a ground truth for the evaluation of segmentation results on the PET image. Various tumour uptake distributions of the irregular and ellipsoidal lesions were modelled for a tumour-to-background ratio (TBR) of 4. These are shown for the irregular lesion on Fig. 2. The different uptake patterns included:

- Homogeneous uptake
- Two-level uptake as described above for the spherical lesions (only used for the irregular lesion)
- Heterogeneous Gaussian smoothed uptake: addition to the background uptake map of a homogeneous uptake smoothed with a Gaussian filter to model higher uptake at the centre
- Necrotic: homogeneous high uptake with no uptake at the centre of the tumour
- Necrotic Gaussian: necrotic uptake smoothed with a Gaussian filter

The phantoms obtained for each case were scanned with an activity concentration in the cartridge of about 6000 kBq/mL, as this provided a PET image with activities corresponding to the original PET scan.

### Evaluation of PET-AS methods

In order to evaluate the performance of state-of-the-art PET-AS methods on heterogeneous target objects of complex geometry, we selected four advanced PET-AS approaches (Table 2) from the recent literature to represent some of the categories described by Bankman et al. [19]. One or more custom implementation of these approaches was written and optimised in house into a common framework using the Matlab package, with the Image Processing Toolbox available for testing. All approaches were implemented as fully automatic 3D algorithms except for WT, since previous work had shown better performance when implemented in 2D [20, 21]. The resulting segmentation methods have been described in more details in the previous work [22]. The clustering approach was implemented for a total number of clusters ranging between 2 and 8, leading to PET-AS methods named GCM2, GCM3, GCM4, GCM5, GCM6, GCM7 and GCM8 in this work. Each of these individual clustering algorithms identifies the lowest intensity cluster as the background and the remaining clusters as the tumour in a final step and provides a single contour for the tumour. This method is used because the aim of the segmentation in this study is to identify the whole lesion outline and because no heterogeneities are modelled in the close neighbourhood of the lesions.

**Table 2:** Description and name of PET-AS methods used in this study. The references correspond to recent publications using similar PET-AS algorithms

Segmentation approach	Name	Description
Adaptive thresholding	AT	3D iterative background-subtracted thresholding
Region-growing	RG	3D iterative region-growing with automatic seed finder
Clustering	GCM2-GCM8	3D fuzzy C-means segmentation with Gaussian mixture modelling, identifying 2, 3, 4, 5, 6, 7 or 8 clusters
Watershed Transform	WT	Slice-by-slice watershed transform-based segmentation, with automatic seed finder

The resulting ten PET-AS methods were applied for all target lesions to the region of the original scan corresponding to an extension of 10-mm margin of the true contour’s bounding box. The segmentation accuracy of each PET-AS was assessed by comparing the contour obtained to the true contour (extracted from the printout template) using the dice similarity coefficient (DSC) [23] which quantifies the similarity between reference and evaluated volume returning a score between 0 and 1. We used a DSC above 0.7 as an indicator of good overlap:

1 $$ \mathrm{D}\mathrm{S}\mathrm{C}=\frac{2*\left|A{\displaystyle \cap }B\right|}{\left|A\right|+\left|B\right|} $$

where *A* is the set of voxels in the reference volume and *B* is the set of voxels in the evaluated volume.

In addition, the sensitivity (*S*) and positive predictive value (PPV) were calculated with the following equations:

2 $$ S=\frac{\mathrm{TP}}{\mathrm{TP}+\mathrm{F}\mathrm{N}}=\frac{A{\displaystyle \cap }B}{A} $$

3 $$ \mathrm{P}\mathrm{P}\mathrm{V}=\frac{\mathrm{TP}}{\mathrm{TP}+\mathrm{F}\mathrm{P}}=\frac{A{\displaystyle \cap }B}{B} $$

with TP the true positives (voxels accurately classified), FN the false negatives (voxels in true contour *A* not included in *B*) and FP the false positives (voxels in contour *B* not included in true contour *A*).

For comparison purposes, the performance of the PET-AS methods was also evaluated using the commonly used NEMA IEC body phantom with spherical plastic inserts. In particular, the results obtained for the irregular lesion which had a volume of 5.9 mL were compared with the segmentation results obtained for the 5.6 mL sphere of the NEMA IEC body phantom scanned at a TBR of 4.

## Results

### Experimental method and reproducibility

#### Printing quality

In the homogeneity test, the number of counts measured with the TLC along the stripes of paper printed in both directions was within ± √ *μ* (with *μ* as the mean value measured). This is in line with a Poisson distribution expected for the decay of ^18^F atoms. The resulting curves followed a horizontal trend, showing that there was no variation in the number of counts across the stripes.

For the 60 ROIs drawn on consecutive slices corresponding to the same homogeneous grey-level square, the average difference to the mean ROI value was 4.2 %, with a variation range of 0.27–12.8 %.

#### Printer calibration

**Fig. 3:**
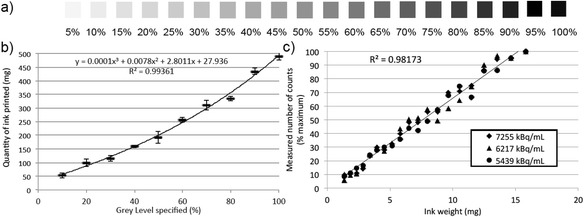
**a** Example of grey-level patterns printed and associated PET image with ROIs, **b** average measured weight of deposited ink and associated standard deviations, **c** average ROI measured counts for printing with black ink and ^18^F-FDG

Figure 3a shows an example of the grey-level pattern printed and scanned in this experiment. Figure 3b shows the non-linear relationship linking the grey levels specified and the amount of ink deposited on the paper when printing with a mixture of black ink and ^18^F-FDG. The curve was best fitted to a third-degree polynomial (*R*^2^ > 0.99). The corresponding equation was used to transform grey-level values specified to the amount of ink deposited on the paper. Figure 3c shows the relationship linking the amount of ink deposited on the paper and the number of counts measured from the grey-level ROIs, for the three activity concentrations considered. The combined data obtained for all activity concentrations showed a good fit to a linear curve (*R*^2^ > 0.98).

The error in the position of the alignment markers, measured on the PET images at three different locations in the image, was systematically smaller than 2.3 mm, which corresponds to a displacement of one voxel. This was expected since the measurements were made on the PET image and were therefore limited by the voxel size. No systematic error was observed.

### Generation of realistic 3D uptake maps

**Fig. 4:**
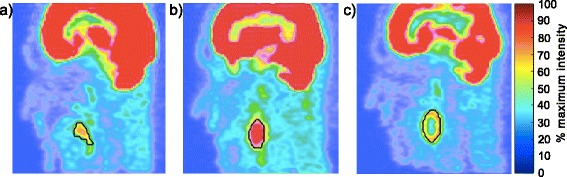
Sagittal view of the images obtained with the printed SS phantom for **a** the irregular homogeneous lesion, **b** the spheroidal homogeneous lesion and **c** the necrotic spheroidal lesion

Figure 4a, b shows a sagittal view of the images obtained with the printed SS phantom modelling a homogeneous irregular and spheroidal H&N lesion, respectively. A total of nine test images were obtained for the spheroidal and irregular lesions modelled with four and five different uptake distributions. Figure 4c depicts a necrotic spheroidal lesion. The corresponding ground truth contour is shown in black.

### Evaluation of PET-AS methods

**Fig. 5:**
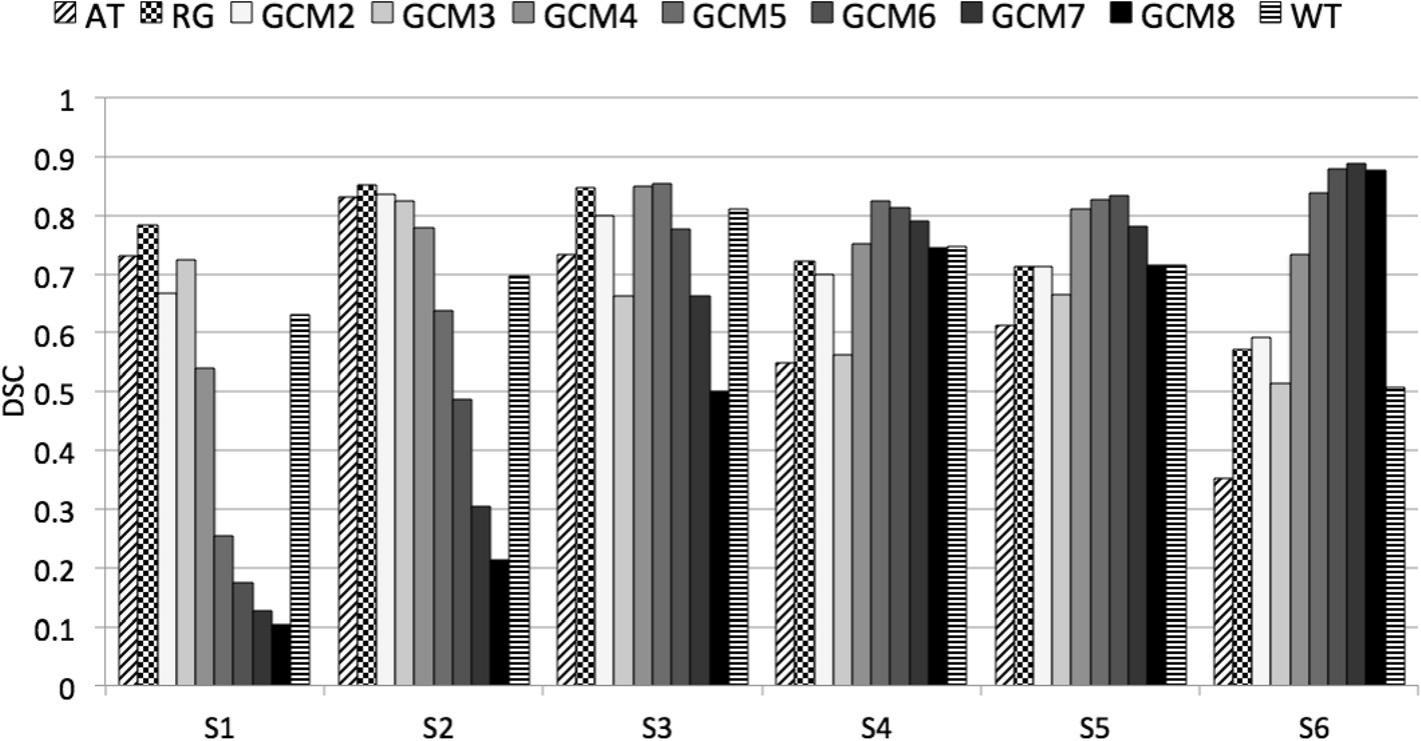
DSC obtained by the PET-AS methods for 6 spheres modelled with a two-level uptake

Figure 5 depicts the DSC values obtained by the different PET-AS methods when delineating spheres S1–S6 modelled with a two-level uptake. The corresponding *S* and PPV are given in Table 3. It can be noticed that binary methods such as AT, RG and WT failed to accurately delineate the largest sphere (DSC<0.6). The DSC values of these binary methods decreased with sphere size, which was correlated to a low *S* value. On the other hand, PPV for these methods was higher than 0.9 for all spheres larger than S2. The GCM method reached DSC values close to 0.9 for S6, when used with 7 clusters. In the case of small spheres, the accuracy of GCM was higher for small numbers of clusters. When increasing the sphere size, the DSC obtained with GCM was gradually higher for larger numbers of clusters. This was due to (a) decreased *S* of methods with small number of clusters and (b) increased PPV with sphere size for methods with larger number of cluster. The optimal number of clusters to use was 3, 2, 5, 5, 6 and 7 for spheres S1, S2, S3, S4, S5 and S6, respectively. Following these results and since the lesion size in the next experiments was smaller than 11.5 mL, we used a maximum of 6 clusters with the GCM method in the rest of the work.

**Table 3:** S and PPV obtained by the PET-AS methods for 6 spheres modelled with 2 uptake levels

	S1		S2		S3		S4		S5		S6	
	S	PPV	S	PPV	S	PPV	S	PPV	S	PPV	S	PPV
AT	0.625	0.882	0.771	0.902	0.579	1.000	0.379	1.000	0.443	0.995	0.214	1.000
RG	0.833	0.741	0.833	0.870	0.748	0.976	0.564	1.000	0.562	0.978	0.400	1.000
GCM2	0.583	0.778	0.792	0.884	0.692	0.949	0.546	0.969	0.560	0.978	0.421	1.000
GCM3	0.708	0.739	0.729	0.946	0.495	1.000	0.392	1.000	0.502	0.983	0.346	1.000
GCM4	0.833	0.400	0.875	0.700	0.794	0.914	0.617	0.959	0.715	0.936	0.581	0.994
GCM5	0.917	0.148	0.938	0.484	0.953	0.773	0.780	0.876	0.806	0.850	0.740	0.969
GCM6	0.958	0.096	0.958	0.326	0.991	0.639	0.868	0.764	0.921	0.761	0.845	0.915
GCM7	0.958	0.068	0.979	0.181	1.000	0.495	0.925	0.689	0.964	0.657	0.906	0.870
GCM8	1.000	0.055	1.000	0.119	1.000	0.334	0.952	0.612	0.992	0.558	0.951	0.814
WT	0.750	0.546	1.000	0.533	0.757	0.871	0.608	0.965	0.600	0.884	0.346	0.942

**Fig. 6:**
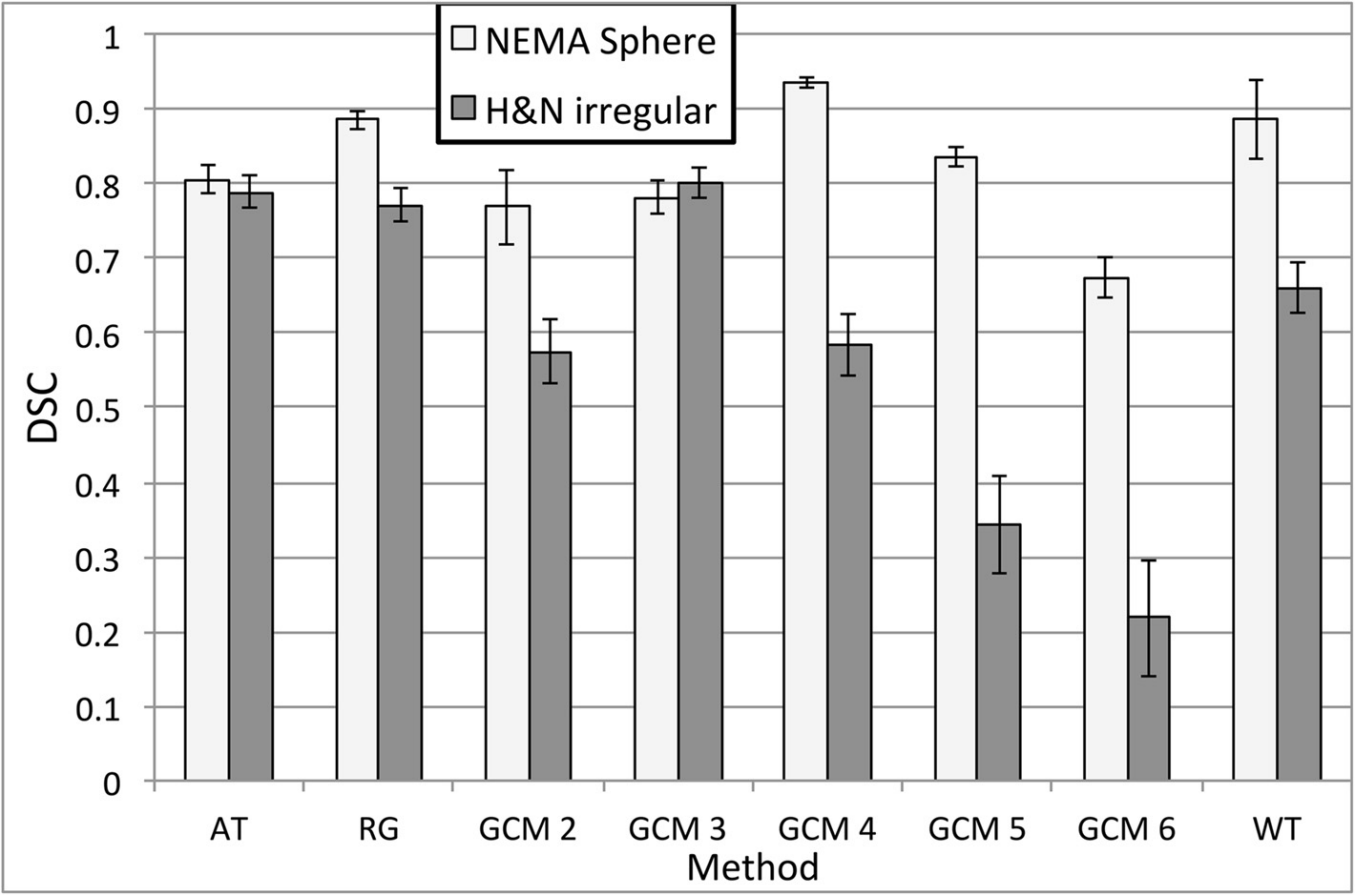
Comparison of DSC values obtained for each PET-AS method tested on the regular NEMA sphere S5 and H&N irregular lesion of same volume. The error bars represent an estimate of the effect of the experimental error on DSC

Figure 6 shows the accuracy (DSC) obtained by the different PET-AS methods listed in Table 2 when delineating the irregular lesion modelled with the printed SS phantom, with the results obtained for the 5.6 mL sphere of the NEMA IEC body phantom shown for comparison. The error bars represent the estimated error on the DSC due to errors in the experimental setup. In particular, the reproducibility error in the measurement of the activity injected in the phantom or the cartridge was within 2 % of the true value according to standard calibration test carried out in our centre. Consequently, the error bars were derived as ±4 % of the value of (1−DSC), to account for the fact that the most accurate methods are expected to be the least sensitive to variations in the TBR and image quality. Lower accuracy was obtained for the irregular lesion compared to the NEMA sphere for all methods except GCM3. Differences were larger than the 4 % error estimate for all methods except AT and GCM3, with the largest differences observed for the remaining clustering (GCM) methods and WT (68 % difference). The accuracy of GCM versions peaked for an optimal number of clusters, which was 4 in the case of the NEMA sphere and 3 for the irregular lesion.

**Fig. 7:**
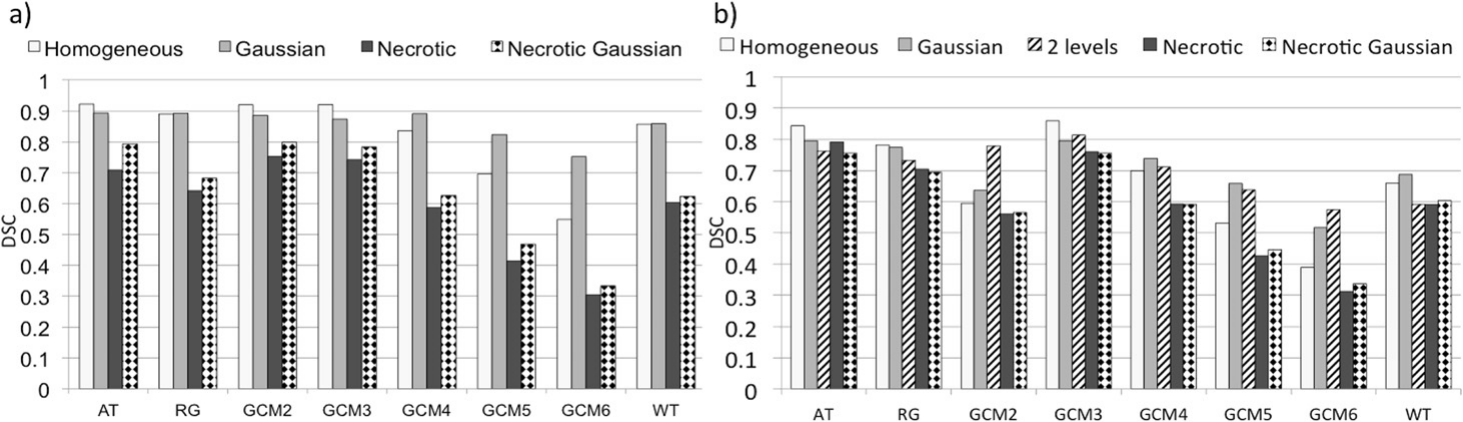
DSC obtained by the PET-AS tested with different uptake patterns for **a** the spheroidal lesion and **b** the heterogeneous lesion

Figure 7a shows the DSC values obtained by the different PET-AS methods for the spheroidal lesion. The corresponding *S* values and PPV are given in Table 4. For the non-necrotic uptake distributions (homogeneous and Gaussian), DSC values were within 5 % of each other for all methods except for GCM with more than 3 clusters. The DSC values for non-necrotic uptake obtained by AT, RG, GCM2 and GCM3 were also within 5 % of each other and within 10 % of the values obtained by WT. These high DSC values (DSC>0.8) were linked to *S* values higher than 0.9 for WT, PPV values higher than 0.9 for AT, and PPV and *S* values just below 0.9 for RG. GCM methods had increasing *S* and decreasing PPV with an increasing number of clusters. For necrotic lesions, differences between DSC values reached by the different methods were as high as 25 %. The *S* for necrotic lesions was higher than 0.9 for the necrotic uptakes, with a PPV lower than 0.7 for all methods except AT. The accuracy of GCM versions peaked at 3, 4 and 2 clusters for homogeneous, Gaussian and necrotic uptakes, respectively. The difference between DSCs obtained by the different GCM methods was largest for necrotic uptakes and smallest for the Gaussian uptake.

**Table 4:** S and PPV obtained by the PET-AS methods for the spheroidal and irregular H&N lesion for different uptake patterns (cf. Fig. 7)

	Homogeneous		Gaussian		Two-level		Necrotic		Necrotic Gaussian	
	S	PPV	S	PPV	S	PPV	S	PPV	S	PPV
	Spheroids									
AT	0.845	1.000	0.852	0.939	N/A	N/A	0.814	0.626	0.922	0.696
RG	0.891	0.889	0.892	0.893	N/A	N/A	0.998	0.473	1.000	0.518
GCM2	0.795	1.000	0.791	1.000	N/A	N/A	0.924	0.635	0.983	0.673
GCM3	0.854	0.996	0.817	0.940	N/A	N/A	0.956	0.605	0.998	0.645
GCM4	0.952	0.745	0.911	0.873	N/A	N/A	0.996	0.416	1.000	0.455
GCM5	0.979	0.539	0.975	0.713	N/A	N/A	1.000	0.261	1.000	0.306
GCM6	0.998	0.378	0.992	0.605	N/A	N/A	1.000	0.179	1.000	0.200
WT	0.962	0.773	0.954	0.780	N/A	N/A	0.956	0.440	0.979	0.539
	Irregular lesions									
AT	0.963	0.751	0.755	0.843	0.961	0.632	0.969	0.670	1.000	0.608
RG	0.994	0.645	0.810	0.742	0.993	0.581	1.000	0.543	1.000	0.533
GCM2	1.000	0.423	0.982	0.472	0.954	0.659	1.000	0.389	1.000	0.395
GCM3	0.976	0.768	0.767	0.828	0.934	0.721	0.994	0.616	1.000	0.608
GCM4	1.000	0.538	0.896	0.629	0.980	0.558	1.000	0.421	1.000	0.420
GCM5	1.000	0.362	0.976	0.497	0.993	0.470	1.000	0.270	1.000	0.287
GCM6	1.000	0.242	1.000	0.348	1.000	0.403	1.000	0.185	1.000	0.203
WT	0.988	0.495	0.865	0.571	1.000	0.420	1.000	0.419	0.994	0.433

Figure 7b shows the DSC values obtained by the different PET-AS methods tested for the segmentation of the irregular lesion. *S* values and PPV are shown in Table 4. Large differences in accuracy between PET-AS methods are visible, with AT performing 8 and 22 % better than RG and WT, respectively, for homogeneous uptake. Again, the DSC values reached for the GCM methods varied between the different versions implemented for 2 to 6 clusters. This effect was larger than for spheroidal lesions, particularly for non-necrotic uptakes, and was largest for necrotic uptakes. Method GCM3 achieved the highest DSC for all uptake distributions. The *S* was high (*S*>0.9) for all uptakes except the Gaussian uptake. PPVs were remarkably lower than for the spheroidal lesion, except for GCM3, and were particularly low for binary methods for highly heterogeneous (two-level and necrotic) uptakes. The largest drop in DSC between the lesions of 31 % of the value for the spheroid was obtained among the binary methods for WT for Gaussian uptake. For the GCM methods, the largest drop in DSC between the lesions was 35 % obtained for GCM3 for homogeneous uptake.

**Fig. 8:**
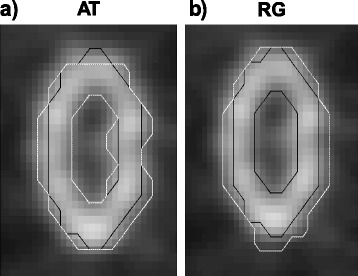
Result of the segmentation (*white*) of a necrotic spheroidal lesion for **a** AT and **b** RG. The black line corresponds to the reference contour

Figure 8 illustrates the fact that different methods included (RG and GCM2-6) or did not include (AT and WT) the necrotic area in the segmented contour for spheroidal lesions. This is shown with the examples of methods AT and RG. Method RG, which did include the necrotic region in the delineated volume, reached 9 and 14 % lower DSC than AT for necrotic and necrotic Gaussian uptakes, respectively, in the spheroidal lesion.

## Discussion

This work aimed at evaluating a variety of promising advanced PET-AS methods for segmenting target objects of complex geometry and heterogeneous or necrotic uptake. For this purpose, we have developed a printed SS phantom technique, which allows generating a physical 3D object modelling any desired tracer uptake distribution with a known ground truth, which is the printout template. The feasibility of producing radioactive two-dimensional PET sources by printing a mixture of ink and ^18^F-FDG had been demonstrated by Markiewicz et al. [17] previously. In this work, we have taken this idea forward by generating a 3D object from a large number of printed sheets and showed its usefulness for evaluating the performance of PET segmentation algorithms. We obtained a good homogeneity and reproducibility of the grey-level printing, with the equipment used for this work (cf. 3.A.2.). The technique was also calibrated for the accurate modelling of uptake values, to ensure that the tumour-to-tissue ratios printed corresponded to the values modelled. A non-linear relationship between the intensities specified and those measured on the PET scan was found and accounted for through a parametric calibration curve. This is in line with the observation made by Markiewicz et al. with different equipment [17], but in addition, we have also shown that this effect is due to the non-linear deposition of ink on the paper. The use of radioactive cross-marks printed on the paper allowed good alignment of the printouts, with small measured errors likely to be due to noise in the PET image obtained. The distance between the markers and the printout was set to 20 mm on average, to ensure that the signal from the markers did not affect the signal from the lesions or neighbouring background. The protocol and calibration procedure described in this work may be applicable to other equipment. The amount of time necessary for a single operator to prepare the phantom was small enough to allow scanning the phantom within one half-life of the ^18^F decay. The total exposure to the radioactive tracer for one session with a single scan was comparable to the exposure of manipulating a conventional fillable phantom.

Our phantom technique allowed modelling non-spherical target objects and large heterogeneities in both background and lesion, which would not have been possible in a controlled and reproducible way with a standard fillable phantom. The examples of PET images obtained given on Fig. 4 show that realistic H&N uptake modelling was achieved, without using walls to separate lesion and background ^18^F-FDG uptakes as in fillable phantoms. In this work, phantom production was limited to oropharynx tumours. However, a printout template could easily be derived for any other site of the body for which a CT scan is available. The printout could also be made more realistic by including a larger number of ^18^F-FDG uptake levels if needed.

The 15 PET scans of the printed SS phantom including both regular and irregular lesions modelled with different uptake patterns allowed a systematic evaluation of the advantages and disadvantage of the different PET-AS algorithms. Binary methods AT, RG and WT, as well as GCM2 clearly lacked sensitivity for the highly heterogeneous spheres (cf. Fig. 5 and Table 3). In these heterogeneous cases, the methods only delineated the high uptake level in the lesion. This can be sufficient when delineating a tumour subvolume for dose escalation. However, our data shows that multiple clustering methods may be preferred when delineating the whole PET-avid tumour. This lack of sensitivity was not observed for the irregular lesion, even when modelled with two uptake levels. In this case, the smaller size of the lesion and its irregular contours had a larger impact than the heterogeneous uptake and led to high *S* values and low PPVs for the binary methods (cf. Table 4).

Table 3 also showed that GCM increased in sensitivity (and decreased in PPV) with increasing numbers of clusters, which was observed for spheroidal and irregular modelled H&N lesions as well (cf. Fig. 7 and Table 4). This is due to the inclusion of more clusters in the tumour contour when a higher number of clusters is used in the algorithm. Our results are in line with work by Hatt et al. [2] which showed the superiority of their clustering algorithm using 3 clusters compared to binary segmentation in the case of heterogeneous lesions. Hatt et al.’s method still differs from GCM in that it uses fuzzy levels and a variety of cluster intensity distribution models, which may explain their use of only 3 hard classes. The images obtained with our printed SS phantom showed the need to use of a number of clusters higher than 2 for heterogeneous lesions to delineate the whole tumour and allowed us to identify the optimal number of clusters to apply in different cases.

The comparison between the segmentation of the irregular lesion modelled with homogeneous uptake and the sphere from the NEMA IEC body phantom (cf. Fig. 6) showed a visible decrease in performance of all segmentation methods. This can be explained by the more complex geometry and by the absence of plastic walls in the printed SS phantom. In fact, we have previously shown that inactive walls lead to a lower activity recovery [9, 20, 24] and can influence the accuracy of image segmentation. The comparison of spheroidal and irregular lesions (cf. Fig. 7, Tables 3 and 4) showed that larger differences in accuracy between methods as well as between uptake patterns for the same method could be observed when testing the method on the smaller and irregular lesions. Our data also highlighted the robustness of the AT method to lesion geometry (Fig. 6) and to necrotic areas in the tumour (Fig. 7, Tables 3 and 4) compared to the other binary methods. This may be due to the fact that AT does not include any spatial connectivity aspect in the segmentation, compared to methods using the region-growing process which penalises complex shapes for RG and WT.

In the case of large necrotic lesions, our results showed that some PET-AS methods generated a volume enclosing the central necrotic region in the final contour, while others (AT and WT) did not include this region, and considered it as part of the background (cf. Fig. 8). In this work, we decided not to include the necrotic volume in the ground truth contour and evaluated the performance of the PET-AS algorithms accordingly. Although no uptake was modelled in the necrotic area, the PET intensity was similar to the background intensity due to noise and spill-out effects. This led to low PPV for the methods including the necrotic area, while PPV for AT remained above 0.6 (cf. Table 4). For RG, this can be explained by the growing process used with one seed only, searching for neighbouring voxels in all directions, and making the method unable to delineate annular shapes. GCM used with more than 3 clusters also included the necrotic area, because the large number of clusters, inadequate for such a homogeneous tumour (when excluding the necrotic centre), makes it likely to add low uptake regions to the tumour.

It should be noted that although we covered a wide range of different segmentation methods, more advanced PET-AS algorithms could be evaluated using the printed SS technique presented in this work. In particular the use of image pre-processing tools to denoise and deblur PET images as suggested by Geets et al. [25] and the application of other recently published promising methods such as GMM [26] and FLAB [27, 28] could provide and even more exhaustive set of data in evaluating the performance of PET-AS methods in delineating heterogeneous and irregular lesions.

This study was conducted using the acquisition and scanning parameters routinely used for clinical scans at our centre so that the results could be readily applicable to routine clinical practice. Parameters such as image noise, reconstruction voxel size, post-filtering and TOF correction have been shown in previous studies [29, 30] to have a potentially important impact on image segmentation. Since this work mainly focused on the use of a novel printed SS phantom technique to produce realistic heterogeneous and irregular lesions, we did not evaluate the dependence of the performance of each PET-AS method with image noise and other parameters used in image reconstruction. This topic could be the subject of future work using the printed SS phantom technique.

The flexibility in the design of ^18^F-FDG uptake patterns provided by the printed SS phantom allowed lesions to be represented with any geometry or uptake distribution, modelling heterogeneities, necrotic regions and, theoretically, microscopic tumour extension. Our work has shown the information that can be extracted using such images compared to homogeneous spherical uptake images. This is a key advantage, in the light of recent studies showing the high impact of segmentation inaccuracies on the dosimetry during radiotherapy treatment in the case of heterogeneous or low intensity lesions [4]. The printed SS phantom technique could be used for many other applications beyond the evaluation of PET segmentation algorithms, such as the assessment and characterisation of combined PET and computed tomography (CT) scanners and the investigation of PET-reconstruction and post-processing methods. Although 3D printing of hollow objects has been used to produce patient-specific plastic inserts [31], such techniques did not provide any flexibility in modifying the phantom and do not allow modelling any heterogeneity as was done in this study. In addition, the printed SS phantom does not use any physical separation (i.e. plastic walls) between the model tumour and background uptake in the transverse plane, which makes it again more realistic than the use of fillable inserts. Although we have shown that the printed SS phantom can be extremely useful in generating realistic target images for segmentation evaluation purposes, the current technique may not yet be adequate for fully quantitative studies. The presence of plastic sheets limits the modelling to details larger than 2 mm in superior-inferior direction, and the scatter and attenuation properties of the plastic, which is the main material in the phantom, do not currently allow modelling human tissue appropriately. The use of a 3D printer to generate PET phantoms was investigated by Miller et al. [32], but the authors acknowledge that the technique does not currently allow printing non-uniform areas of tracer uptake. Work is in progress at our centres to further develop the technique to make it applicable to other quantitative studies.

## Conclusion

This work presents a novel phantom technique for the evaluation the performance of PET auto-segmentation methods in delineating heterogeneous and irregular lesions. We developed a method to print a subresolution sandwich phantom with radioactive ^18^F-FDG maps. We have shown that our method can be successfully used to design, print and acquire PET images of complex and realistic H&N uptake with ground truth data. We have also demonstrated the usefulness of the printed subresolution sandwich phantom technique in assessing the performance of advanced PET automatic segmentation methods when delineating target objects with highly heterogeneous uptake and complex geometry. The printed subresolution sandwich phantom technique has the potential of playing a key role in future 3D quantitative methods in radionuclide imaging.
